# Beyond the bladder: case series of *Mycobacterium bovis* bacillus Calmette-Guérin infections following intravesical instillation

**DOI:** 10.1128/asmcr.00207-25

**Published:** 2026-05-21

**Authors:** Adrienne A. Workman, Ourania Parra, Nicole A. Loeven, Prabhjot Kaur, Florian R. Schroeck, John D. Seigne, Rebecca Wang, Isabella W. Martin

**Affiliations:** 1 Department of Pathology and Laboratory Medicine, Dartmouth Hitchcock Medical Center 826973, Lebanon, New Hampshire, USA; 2 Department of Pathology, Boston Children's Hospital 571184, Boston, Massachusetts, USA; 3 Department of Surgery, Section of Urology, Dartmouth Hitchcock Medical Center 207077, Lebanon, New Hampshire, USA; 4 White River Junction VA Healthcare System 20127, White River Junction, Vermont, USA; 5 The Dartmouth Institute for Health Policy & Clinical Practice 539576, Lebanon, New Hampshire, USA; 6 Section of Infectious Diseases and International Health, Department of Medicine, Dartmouth Hitchcock Medical Center 200888, Lebanon, New Hampshire, USA; Pattern Bioscience, Austin, Texas, USA

**Keywords:** BCG, *Mycobacterium bovis*

## Abstract

Intravesical instillation of *Mycobacterium bovis* bacillus Calmette-Guérin (BCG) was first used to treat bladder cancer in 1976. Since then, it has become a common therapy for non-muscle invasive bladder cancer. A live, attenuated mycobacterium, *M. bovis* BCG causes infectious complications in approximately 1% of patients. These infections can involve various anatomic sites and have a range of presentations. Diagnosis may require several testing modalities, and treatment typically involves multiple antimicrobials for a long duration. We report three cases of *M. bovis* BCG infection following intravesical BCG instillation for the treatment of bladder cancer. The patients are men ranging in age from 71 to 83 years old. One had a ruptured mycotic aneurysm of the aorta, another had a presumed anaphylactic reaction, and the final had a bone marrow infection. Isolation of BCG was achieved through mycobacterial blood culture in two cases and also through mycobacterial culture of bone marrow and hematoma aspirate. Nucleic acid amplification testing for *Mycobacterium tuberculosis* complex (of which BCG is a member) was utilized in two cases, and histologic examination identified granulomas in one case. Two of the patients received mycobacterial treatment, including rifampin, isoniazid, ethambutol, and linezolid. One patient remains living over 3 years after infection, one died despite treatment, and one died from unrelated causes. These cases highlight the diversity of BCG infection symptoms and sites and the various laboratory testing modalities that can inform diagnosis and treatment.

## INTRODUCTION

*Mycobacterium bovis* is a member of the *Mycobacterium tuberculosis* complex alongside *M. tuberculosis sensu stricto* and others. A culture-adapted, attenuated strain of *M. bovis*, named *M. bovis* bacillus Calmette-Guérin (BCG), was developed in the early 1900s for use as a live vaccine to prevent *M. tuberculosis* infection and first used in 1921. Since then, BCG has become one of the most administered vaccinations in the world (1, 2).

Around the same time, an autopsy study documented that active tuberculosis lesions were found less often in deceased patients with malignancy than in those without (3). A hypothesis developed that exposure to the BCG vaccine may convey an immune reaction to tumor cells. This was tested by Old and colleagues in 1959 when they inoculated mice with BCG then implanted tumor cells. They observed that BCG-inoculated mice developed smaller tumors and had longer survival times than those which did not receive BCG (4). Immunotherapy with BCG continued to be tested in animals, and it was recognized that the best response was achieved with direct contact between BCG and tumor cells (5).

The anatomic accessibility via cystoscopy of superficial bladder tumors makes this tumor type an ideal target for BCG immunotherapy. Morales and colleagues were the first to use intravesical instillation of BCG to treat bladder cancer in 1976 (6). In the decades since, intravesical instillation of BCG has become a common adjuvant therapy for non-muscle invasive bladder cancer, with rates of use increasing in recent decades. BCG instillation has been found to reduce the rate of recurrence and progression when given after transurethral resection of superficial bladder cancer (7–9). The American Urological Association Clinical Practice Guideline for the treatment of non-muscle invasive bladder cancer recommends a 6-week BCG induction course for intermediate- and high-risk patients following transurethral resection of the tumor, consisting of weekly 50 mg intravesical BCG instillations (10). In patients who have persistent disease, a second induction course may be considered (10). In patients with complete response to induction, maintenance therapy of three weekly intravesical instillation of 50 mg BCG is recommended for intermediate-risk patients for 1 year and for high-risk patients for 3 years (10).

Despite the efficacy of BCG for the treatment of non-muscle invasive bladder cancer, infectious complications of BCG instillation occur in approximately 1% of patients (11, 12). These infections may occur over a year after BCG instillation, have variable presentation and anatomic site, and can, therefore, be difficult to diagnose. Importantly, diagnostic methods routinely available in clinical microbiology laboratories do not distinguish between BCG and other members of the *M. tuberculosis* complex. We present three cases of BCG infections at a single institution following intravesical BCG instillation. We discuss presentation, microbiological testing, and treatment of BCG infections. Our institutional review board reviewed this case series study and deemed it “not human research.”

## MATERIALS AND METHODS

Cases of BCG infection were identified through review of the microbiology clinical consultation log at Dartmouth Health. This log includes surgical pathology cases with clinical microbiologist review, requests for select send-out infectious disease testing, and other requests for consultation. All identified BCG infection cases from 2021 to 2024 were included. The laboratory information system and electronic medical record were then reviewed to obtain pertinent demographic, clinical, and testing data for each case.

Testing modalities utilized for identifying mycobacterial infections in these cases included mycobacterial blood and tissue cultures, *M. tuberculosis* complex nucleic acid amplification testing (NAAT), acid-fast bacilli (AFB) staining of formalin-fixed, paraffin-embedded (FFPE) tissue specimens, and whole-genome sequencing.

The mycobacterial blood (BD BacTec Myco/F Lytic culture vials) and tissue cultures were performed at Dartmouth Health in alignment with the *Clinical Microbiology Procedures Handbook* (13). The following specimens and/or testing modalities were sent to various commercial or public health referral laboratories: bone marrow specimen for mycobacterial culture, susceptibility testing of BCG isolates (which included ethambutol, isoniazid, rifampin, and pyrazinamide), and whole-genome sequencing of BCG isolate. The *M. tuberculosis* complex NAAT on a hematoma aspiration sample was performed by Reference Laboratory 1 using a laboratory-developed test that targets the *katG* gene, and the *M. tuberculosis* complex NAAT on FFPE was performed by Reference Laboratory 2 using a laboratory developed test that targets *hsp65*. Susceptibility testing was performed at Reference Laboratory 1 using the BACTEC MGIT 960 platform (Becton Dickinson). AFB staining of FFPE was performed by the histology laboratory at Dartmouth Health.

## RESULTS

**Fig 1:**
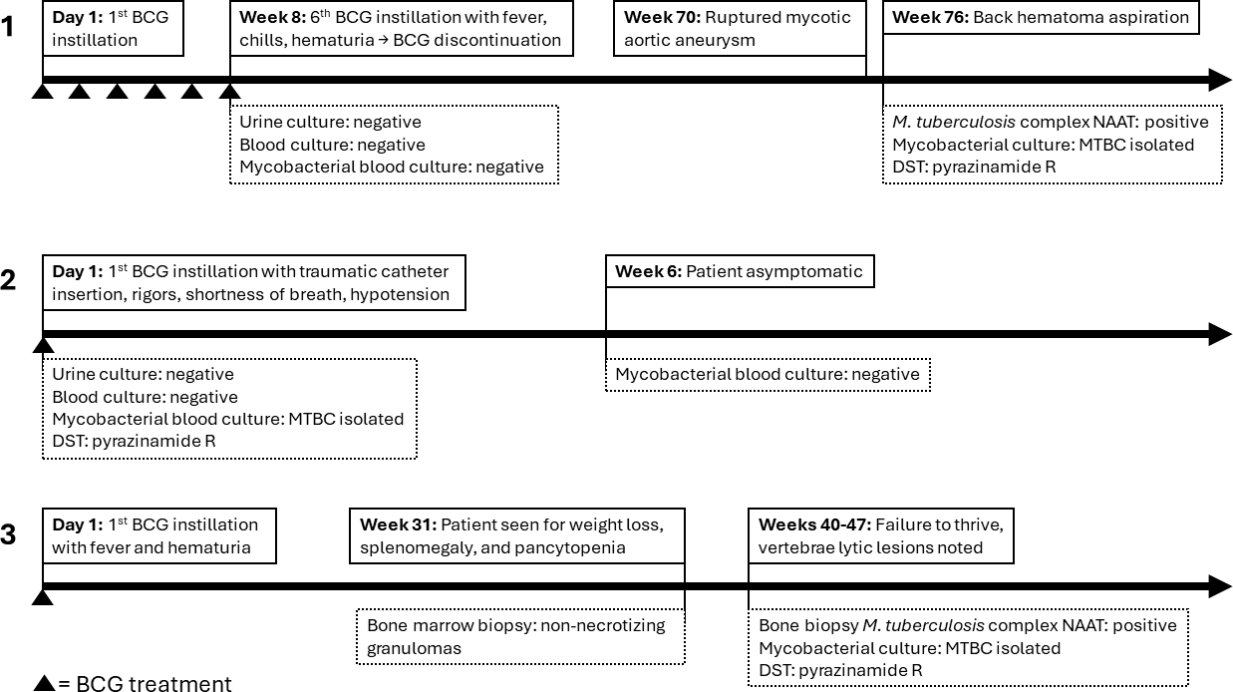
Overview of patient cases 1, 2, and 3 from initial *Mycobacterium bovis* bacillus Calmette-Guérin (BCG) bladder instillation for the treatment of bladder cancer to diagnosis of BCG infection. Boxes with solid lines highlight clinical findings. Boxes with dotted lines highlight relevant laboratory testing, including routine bacterial urine and blood cultures, mycobacterial cultures, *M. tuberculosis* complex (MTBC) nucleic acid amplification testing (NAAT), key drug susceptibility testing (DST) resistance (R), and biopsies.

Three patients with BCG infection were identified from 2021 to 2024 using the microbiology clinical consultation log at Dartmouth Health. The patients were men ranging in age from 71 to 83 years old with histories of intravesical BCG instillation for treatment of bladder cancer. Case details for each of these patients are below (Fig. 1).

### Case 1

A 78-year-old man with recently diagnosed bladder cancer at the end of his first induction course of BCG presented to the emergency department several hours after his sixth BCG treatment with fever, shaking, chills, dysuria, and hematuria. Routine bacterial urine and blood cultures, as well as mycobacterial blood culture, were negative. Due to this reaction, BCG therapy was discontinued and he received alternate therapy for his bladder cancer, including mitomycin C. Approximately 1 year later, the patient suddenly developed severe back pain. Workup revealed a ruptured infrarenal aortic aneurysm. During operative repair, there was concern for a mycotic aneurysm, but no samples were sent for culture or histology. Empiric mycobacterial therapy was initiated with rifampin 600 mg daily, isoniazid 300 mg daily, ethambutol 1,600 mg daily, and linezolid 600 mg two times daily, but the patient developed severe side effects, including acid reflux and fatigue. The treatment was discontinued after 2 weeks, and a plan was made to aspirate a hematoma in his back adjacent to the prior aneurysm for more definitive diagnosis. The aspiration sample both had mycobacterial culture performed in-house and was sent to a reference laboratory for *M. tuberculosis* complex NAAT. The NAAT returned positive for *M. tuberculosis* complex after 5 days, and *M. tuberculosis* complex was isolated from culture after 5 weeks. Susceptibility testing of first-line anti-mycobacterial agents showed the isolate was susceptible to isoniazid, ethambutol, and rifampin but resistant to pyrazinamide. Due to the pyrazinamide resistance and the history of BCG exposure, the isolate was presumed to be *M. bovis* BCG. The patient was again initiated on antimycobacterial therapy, this time with rifabutin 300 mg daily, isoniazid 300 mg daily, ethambutol 1,200 mg daily, and levofloxacin 750 mg daily, though continued to experience side effects. After a year of induction therapy, he was switched to a two-drug regimen, eventually coming to isoniazid 300 mg daily and rifabutin 300 mg daily to achieve the most optimal balance of efficacy and tolerability. He remains alive over 3 years after his aneurysm rupture, and continuation of his antimycobacterial regimen is recommended indefinitely.

### Case 2

An 83-year-old man with a 7-year history of bladder cancer with prior BCG therapy was found to have a high-grade recurrence of his bladder cancer. He underwent a transurethral resection of his bladder tumor then presented a month later for an induction dose of BCG. Initially, the Foley catheter was inserted with return of urine, the balloon was inflated without pain, and the BCG was instilled into the bladder for 30 min. At this time, the patient developed rigors, back pain, and facial erythema. The catheter was removed, and the patient was noted to have gross blood from the meatus. A Foley was reinserted to drain the BCG, and during the procedure, there was a traumatic false pass into the prostatic urethra. The patient developed wheezing, shortness of breath, and tachycardia. Albuterol was given and oxygen was delivered by nasal cannula. After transfer to the emergency department, he also developed hypotension (systolic blood pressure in the 50–60 mmHg range) and altered mental status. There was concern for anaphylactic vs septic shock, and urine, blood, and mycobacterial blood cultures were collected. Multiple doses of intramuscular epinephrine were administered. The infectious disease service was consulted urgently and recommended not initiating therapy for mycobacterial infection. He was admitted to the intensive care unit (ICU) where norepinephrine and epinephrine were administered for ongoing hypotension which resolved over the course of a few hours. Although his white blood cell count while in the emergency department was within normal limits, it peaked at 14.7 × 10^3^/mcL (normal range: 4.0 × 10^3^/mcL–9.5 × 10^3^/mcL) while in the ICU. He returned to his respiratory, hemodynamic, and mental status baseline by the following day, and he was discharged to home with close follow-up. The routine urine and blood cultures were negative, but the mycobacterial blood culture isolated *M. tuberculosis* complex after 22 days. By the time this culture resulted, the patient had recovered and had no further fevers or hematuria. Susceptibility testing of first-line anti-mycobacterial agents showed the isolate was susceptible to isoniazid, ethambutol, and rifampin but resistant to pyrazinamide. The isolate was presumed to be *M. bovis* BCG due to the patient’s history of BCG therapy and the resistance to pyrazinamide. A repeat mycobacterial blood culture was negative, and no mycobacterial treatment was given. The patient died approximately 4 months later from an unrelated metastatic adenocarcinoma.

### Case 3

**Fig 2:**
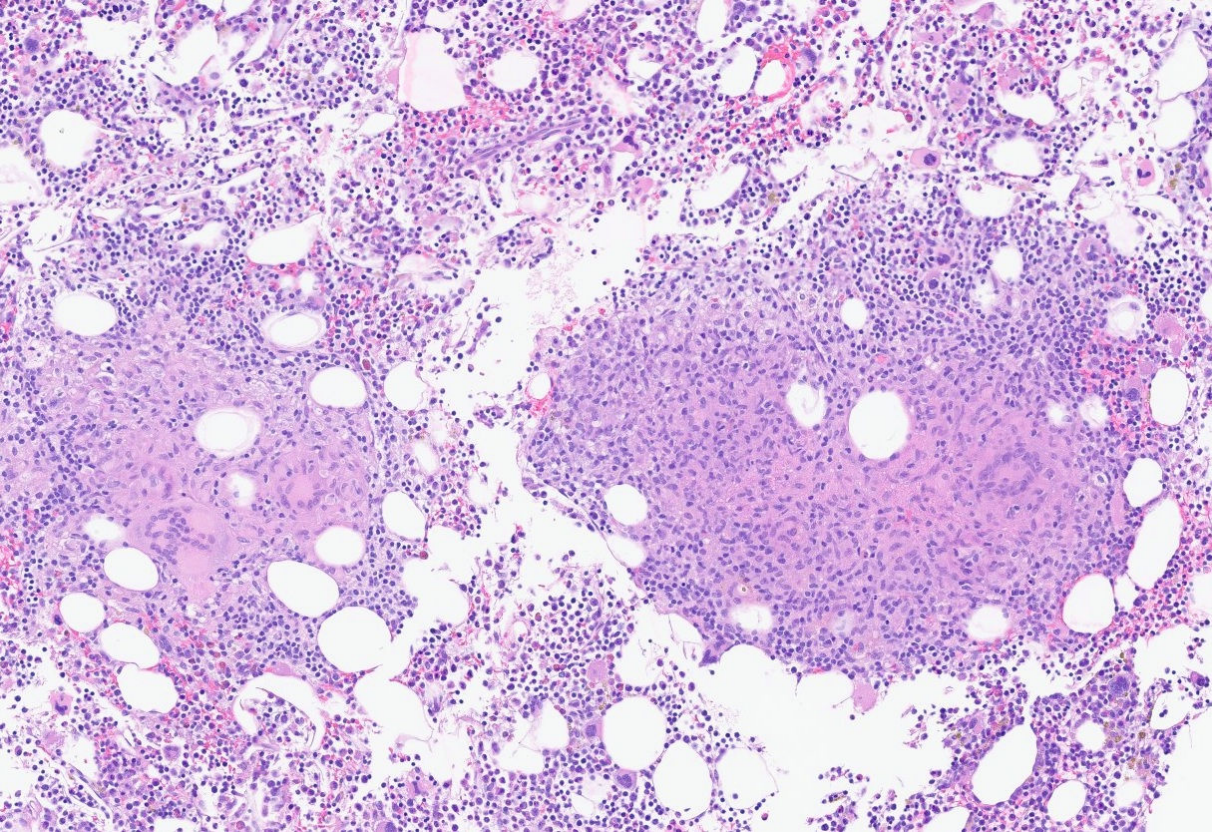
Bone marrow biopsy of a 71-year-old man with pancytopenia and splenomegaly which reveals non-caseating granulomas in a background of trilineage hematopoiesis (H&E stain, 100× magnification).

A 71-year-old man with a history of bladder cancer received intravesical BCG therapy and within hours developed fever and hematuria. These symptoms resolved without intervention, but over the following months, he lost over 50 pounds and was noted to have splenomegaly and pancytopenia. Given concern for a hematologic malignancy, a bone marrow biopsy was performed. The biopsy revealed no hematologic malignancy, but noted non-necrotizing granulomas (Fig. 2). A positron emission tomography (PET) scan found fluorodeoxyglucose (FDG)-avid lytic lesions of the T8 and L1 vertebrae, and a bone biopsy of one of these lesions was obtained. Non-necrotizing granulomas were noted, and although the AFB and Grocott’s Methenamine Silver (GMS) stains were negative, the pathologist recommended further mycobacterial studies due to the history of BCG therapy. With heightened concern for mycobacterial infection, further diagnostic workup was pursued including repeat bone marrow biopsy for mycobacterial culture and histopathology, mycobacterial blood culture, and *M. tuberculosis* complex NAAT of his prior bone marrow sample. The patient was started on empiric therapy with levofloxacin 750 mg every 48 h, ethambutol 1,200 mg daily, rifampin 600 mg daily, and isoniazid 300 mg daily. The *M. tuberculosis* complex NAAT on the prior bone marrow returned positive and soon thereafter the mycobacterial blood culture isolated *M. tuberculosis* complex after 24 days. Mycobacterial culture of the repeat bone marrow isolated *M. tuberculosis* complex after 34 days, and histopathology of the repeat bone marrow biopsy showed further granulomas and also hemophagocytosis. Susceptibility testing of first-line anti-mycobacterial agents showed the isolate was susceptible to isoniazid, ethambutol, and rifampin but resistant to pyrazinamide. Additionally, whole-genome sequencing of the isolate was performed at the California Department of Health which confirmed identification of BCG. After further workup, the patient was diagnosed with hemophagocytic lymphohistiocytosis (HLH) thought to be secondary to the BCG infection. The patient had been admitted for about 4 weeks for workup and initiation of treatment before he was discharged to a rehabilitation facility. He continued to deteriorate despite treatment, and within 2 weeks, he transitioned to hospice care and died.

## DISCUSSION

Overall, BCG immunotherapy has an excellent safety profile, but there are risks to instilling a live, attenuated strain of *M. bovis* into the bladder. Immediate side effects from intravesical BCG instillation are most often local, including cystitis, hematuria, and increased urinary frequency (14). Systemic symptoms, such as fever and malaise, occur less often (14). Infections most often involve the genitourinary tract but may also localize to joints, lungs, blood vessels, and bone marrow (12, 15). In our cases, we observed involvement of the aorta and the bone marrow, as well as a presumed anaphylactic reaction.

With so many possible sites of infection, the presenting symptoms of BCG infection can vary widely. Fever is one of the most common presenting symptoms, but other clinical findings may include weight loss, fatigue, malaise, urinary symptoms, dyspnea, and arthralgia among others (12, 16, 17). Symptoms of BCG infections may manifest days to years after instillation, which can be a barrier for patient-facing providers in connecting the dots between exposure and infection, delaying diagnosis (15–17). Some anatomic sites tend to have earlier presentations, including the lungs, liver, and penis (15, 17). Other locations, such as joints, vessels, kidneys, and testes, tend to result in symptoms after months to years (15, 17). Due to the wide range of possible symptoms, anatomic sites, and timelines, these infections can be difficult to diagnose and may require a high level of clinical suspicion to prompt the necessary microbiological testing.

There are multiple testing methods available that may lead to successful laboratory diagnosis of BCG infection. Due to slow growth of mycobacteria and the relatively short duration of incubation of routine blood and urine cultures, these common tests are usually negative, as in our cases (15, 17). However, specific mycobacterial blood, bone marrow, or tissue cultures, all of which use special media and extended incubation times, enable recovery of mycobacteria. Many laboratories may have previously performed mycobacterial (and fungal) blood cultures using Isolator Tubes (Abbott Laboratories, Abbott Park, IL, USA), which were discontinued in 2023. In the absence of Isolator Tubes, laboratories may consider using BacTec Myco/F Lytic bottles (Becton Dickinson Diagnostic Systems, Sparks, MD, USA) or the VersaTREK system (Thermo Scientific, Waltham, MA, USA) which are both FDA-approved or cleared (18). Manual methods or creation of laboratory developed tests using existing assays may also be considered (18). In our cases, mycobacterial blood cultures were performed using BacTec Myco/F Lytic bottles, which have been found to have comparable sensitivity to the Isolator Tubes (19). In two of our cases, mycobacterial blood culture using BacTec Myco/F Lytic bottles resulted in successful isolation of BCG, one after 22 days and the other after 24 days. Our cases also demonstrated successful isolation of BCG from bone marrow and hematoma aspiration specimens.

Routine mycobacterial identification methods available in most clinical laboratories (e.g., matrix-assisted laser desorption/ionization time-of-flight mass spectrometry or off-label use of the Xpert MTB/RIF assay [Cepheid, Sunnyvale, CA, USA]) are not able to distinguish BCG from other members of the *M. tuberculosis* complex. The initial laboratory report of “*M. tuberculosis* complex” can, therefore, lead to confusion on the part of patient-facing providers and may require education from laboratory staff and/or medical directors about the inclusion of BCG within the *M. tuberculosis* complex. In all patients in our series, clinical suspicion for BCG was high due to history of bladder cancer therapy, even when BCG instillation had occurred as long ago as a year prior.

Susceptibility testing of all isolates in this series demonstrated pyrazinamide mono-resistance, a pattern of resistance that is intrinsic to all *M. bovis* isolates, including BCG strains. This resistance is due to a mutation in the *pncA* gene, which encodes an enzyme that converts pyrazinamide to its active form pyrazinoic acid (20). This pattern of susceptibility testing results can be used as a practical surrogate for confirmation of BCG strains in patients with the appropriate clinical history and lack of other risk factors for tuberculosis. However, best practice remains to confirm the identification of BCG with strain-typing, which is available in the United States through the public health laboratory system. This send-out confirmatory testing occurred in only one of our three cases, an omission that was due, in part, to high clinical suspicion for BCG infection. Acquired antimicrobial resistance to other antimycobacterial agents has been described in BCG (21).

NAAT for *M. tuberculosis* complex DNA can be performed on fresh or FFPE tissue specimens and, although it is generally less sensitive than culture, may be useful in conjunction with mycobacterial cultures to improve speed of diagnosis. There are no FDA-cleared NAATs for rapid detection of *M. tuberculosis* complex on non-sputum samples, so such testing requires validation of an off-label specimen type on the Xpert MTB/RIF assay (Cepheid, Sunnyvale, CA, USA), use of a laboratory-developed test, or send out to a reference laboratory. In biopsy or resection specimens, granulomas may be present and organisms may be seen with an AFB stain. Lack of organisms on AFB stain does not necessarily exclude infection, as seen in one of our cases, and additional testing on the FFPE may still be pursued. As with other mycobacterial infections, the presence of granulomas and/or AFB seen on histopathology using special stains or immunohistochemistry increases the pre-test probability of successful detection of BCG DNA within a tissue specimen (22–24). In two of our cases, tissue samples were sent to a reference laboratory where a laboratory-developed test detected *M. tuberculosis* complex. As mentioned above, the Xpert MTB/RIF assay and many laboratory-developed tests will detect BCG as part of the *M. tuberculosis* complex but will not differentiate it from other members of the *M. tuberculosis* complex. A positive NAAT also must be interpreted with caution due to detection of both live and dead bacteria, meaning that this testing is not appropriate for monitoring of treatment response (25).

Definitive microbiological diagnosis is often sought due to the intensive treatment regimens that BCG infections require. Two of our cases resulted in antimycobacterial treatment, with regimens including isoniazid, ethambutol, rifabutin or rifampin, and linezolid or levofloxacin. One had severe side effects from these medications, including severe nausea thought to be from ethambutol and neuropathy from isoniazid, and he was eventually transitioned to a two-drug regimen. BCG infections are typically treated with multi-drug regimens for several months, similar to how *M. tuberculosis sensu stricto* is treated (26). However, since BCG has intrinsic resistance to pyrazinamide, this agent should not be part of treatment.

Overall, these cases highlight the broad spectrum of clinical presentation and the difficulty of diagnosing and treating BCG infections. Consideration of a BCG infection is warranted when a patient with a history of BCG therapy presents with infectious symptoms. Routine testing of asymptomatic patients for BCG is not recommended due to low diagnostic yield and expected urine positivity in patients with recent BCG instillation. However, patient-facing providers should be aware of the wide variety of presentations and timelines of BCG infections in order to pursue mycobacterial testing in symptomatic patients with relevant history of BCG instillation as part of bladder cancer therapy. Depending on the clinical scenario, multiple testing modalities may need to be pursued for diagnosis. Definitive diagnosis may take a long time due to slow growth in culture and send out testing, and in severe cases with high clinical suspicion empiric treatment may be pursued. Laboratories should aim for clear communication with reporting, as reports of *M. tuberculosis* complex, of which BCG is a member, may cause confusion. It is recommended to confirm cases of BCG with strain-typing through public health reference laboratories for documentation of identity and to allow tracking of these cases.

### Conclusion

Intravesical BCG instillation is a common adjuvant therapy for superficial bladder cancer that can rarely result in systemic BCG infections. These infections have widely variable presentations, sites, and timelines, and they can be difficult to diagnose and treat. Various testing methods can aid in the diagnosis of disseminated BCG infection, and as seen in our cases, pursuit of multiple testing modalities may be pursued concurrently to optimize diagnostic yield.
